# Retesting for genital *Chlamydia trachomatis* among visitors of a sexually transmitted infections clinic: randomized intervention trial of home- versus clinic-based recall

**DOI:** 10.1186/1471-2334-13-239

**Published:** 2013-05-24

**Authors:** Hannelore M Götz, Mireille EG Wolfers, Ad Luijendijk, Ingrid VF van den Broek

**Affiliations:** 1 Department of Infectious Disease Control, Rotterdam Rijnmond Public Health Service, Rotterdam, the Netherlands; 2 Department of Medical Microbiology and Infectious Diseases, Erasmus MC Rotterdam, Rotterdam, the Netherlands; 3 Epidemiology & Surveillance Unit, Centre for Infectious Disease Control, National Institute of Public Health and the Environment, Bilthoven, the Netherlands; 4 Department of Public Health, Erasmus MC, University Medical Center, Rotterdam, the Netherlands

**Keywords:** Chlamydia trachomatis, Screening, Repeated infection, Retest rate, Chlamydia positivity, Intervention retesting, Home-based testing versus clinic testing

## Abstract

**Background:**

Reinfections of *Chlamydia trachomatis* (Ct) are common. In a two-armed intervention study at an urban STI clinic in the Netherlands, heterosexual Ct-positive visitors received an invitation for retesting after 4–5 months. Interventions were either home-based sampling by mailed test-kit, or clinic-based testing without appointment.

**Methods:**

Data collection included socio-demographic and sexual behavioural variables at first (T0) and repeat test (T1). Participation in retesting, prevalence and determinants of repeat infection among study participants are described and compared with findings from non-participants.

**Results:**

Of the 216 visitors enrolled in the study, 75 accepted retesting (35%). The retest participation was 46% (50/109) in the home group versus 23% (25/107) in the clinic group (p = 0.001). Men were less often retested than women (15% versus 43%, p < 0.001). The overall chlamydia positivity rate at retest was 17.3% (13/75) compared to 12.4% seen at all visits at the STI clinic in 2011. Repeated infections were more frequent among non-Dutch than Dutch participants (27.0% versus 7.9%; p = 0.04) and in persons reporting symptoms (31.0% versus 7.0%; p = 0.01). Both untreated infections of current partners as well as unprotected sex with new partners contribute to repeated infections.

**Conclusion:**

The high rate of repeated infections indicates the need for interventions to increase retesting; improvement of partner-management and risk reduction counselling remain necessary. Home- based testing was more effective than clinic-based testing. However other strategies, including self-triage of patients, may also increase repeat testing rates and personal preferences should be taken into account.

## Background

Repeat infections of *Chlamydia trachomatis* are frequent and challenging to control [1-4]. Repeat infections increase the probability to develop PID and other complications. Recently, in a population based systematic screening project in Amsterdam, Rotterdam and South- Limburg, infected persons at first test automatically received a repeat testkit after 6 months, based on the advice of a previous pilot-screening project [5]. Sixty-six percent accepted retesting and the proportion with repeat infections was twice the chlamydia positivity at first test [6]. In this Chlamydia Screening Implementation programme (CSI), invitations were sent by postal service and further communication was via the internet; participants received test-packages at home, both for initial tests and retests. In Rotterdam, baseline chlamydia positivity (5.1%) and repeat infection rates (11.3%) were higher than in the other regions [6].

National guidelines in the Netherlands recommend annual rescreening of *Chlamydia trachomatis* infected patients [7]. However, the guidelines do not address retesting of positives and the way to achieve retesting in routine care. Along with STI care in general practice (GP), people at high risk for STI have access to free testing at STI clinics of public health services [8]. The repeat infection rate at STI clinics is not included in the Dutch national surveillance programme, although a recent study in 2012 showed a 19% repeat infection rate in an STI clinic in South Limburg [9]. Repeat testing is a timely way to detect re-infections (resulting from incomplete treatment of index or partner or unprotected sex between the same partners before infection clearance). It can improve the detection of repeated, newly acquired infections in a group with a proven high-risk profile for chlamydia. However, uptake of repeat testing is generally low and the most effective way to increase the uptake is unknown. Guy et al. performed a systematic review and identified posting testkits as an important strategy to increase repeat testing uptake [10].

Our aim was to compare participation and chlamydia positivity at retest using two different recall methods: (1) offering home-based test-kits versus (2) sending personal invitations for retesting at the clinic. We aimed to assess demographic and behavioural factors determining participation in retesting as well as determinants for repeat infection and compare these to available data on routine care at the STI clinic.

## Methods

### Study population

Our prospective Chlamydia Retest Implementation (CRI) study was carried out in heterosexual men and women at an STI clinic with a genital *Chlamydia trachomatis* infection, with whom the risk of re-infections was discussed at the treatment consultation. Patients were asked for consent to participate in a study to facilitate retesting, emphasizing that participants would have personal health gain. We excluded patients with PID, pregnant women, patients with allergies to or other contra-indications for the first choice treatment (Azithromycine), clients under 16 years, and men who have sex with men, because for the latter group the advice is bi-annual screening for all STI. Furthermore, patients with clinically evident signs of infection who receive antibiotic treatment for chlamydia at first visit, i.e. men with symptomatic urethritis or with leucocyturia, and patients notified by a current sexual partner were also excluded, because they did not come back for consultation when the diagnosis was confirmed.

### Study procedures

Two recall methods for retesting were compared: (1) the ‘clinic group’ was asked to visit the STI clinic for retesting without appointment and were given a personal testkit at waiting at the counter (i.e. an envelope prepared for them with sampling tube for urine collection for men or vaginal swab for women); (2) the ‘home group’ were sent a similar testkit to their home address (or other address of choice), which could be sent back free of charge to the laboratory directly. Testing for *Chlamydia trachomatis* was carried out by NAAT (BD VIPER SDA, Becton-Dickinson, New Jersey).

**Figure 1:**
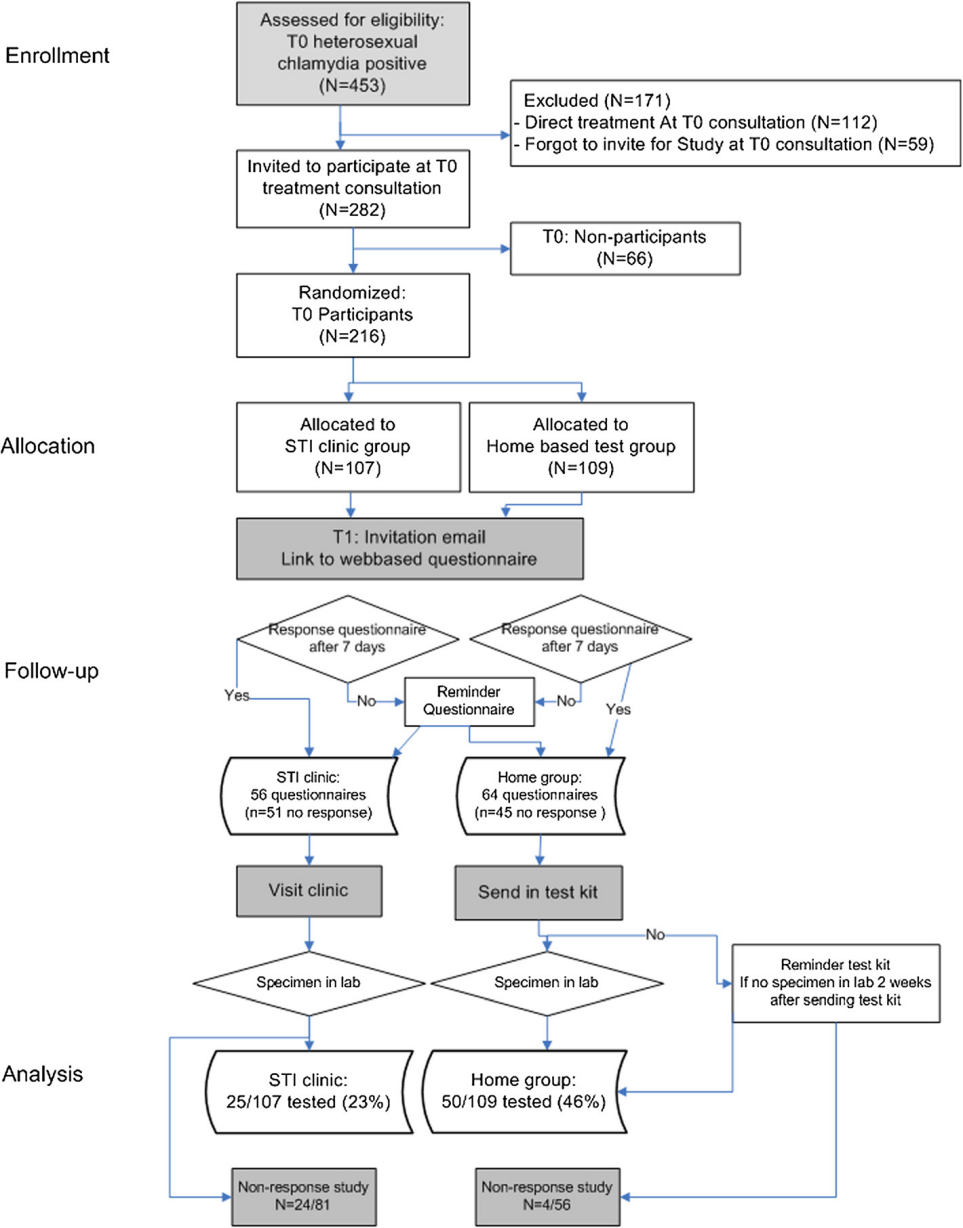
Flow chart of patient enrolment and follow-up of the Chlamydia Repeat test Implementation study and results on participation and positivity.

Patients who had given written consent for the CRI study at baseline (participants) were entered in a database and CRI participants completed a web-based questionnaire at the clinic and provided an email address. Computerized randomisation into home group (home-based testing) or clinic group (STI clinic-based testing) was done using an anonymous list of ID numbers and sex of participants. Enrolled patients were invited for repeat testing 4–5 months after treatment at T0. Using a list of participants, the research nurse sent invitations by email at T1 with information about the retesting method and a second questionnaire. People who did not respond to the initial invitation received a reminder email within 7 days. A reminder was also e-mailed to persons in the home group whose samples were not received within 2 weeks. Responders received their result by SMS and were invited for treatment at the clinic if positive. In case the reminders did not result in retesting after 4 weeks, a questionnaire on reasons for non-response was e-mailed. For an overview see flowchart (Figure 1). The Medical Ethical Committee of the Erasmus University of Rotterdam approved of the study.

### Sample size calculation

The sample size was determined by the number of people positive for chlamydia and the proportion consenting to participate. Anticipating 100 positives per month (average first 6 months 2011), and an initial participation/consent rate of 70%, we expected to be able to recruit 280 participants in a study period of 4 months. This would provide the two groups of 124 persons needed to compare the response rate between the two interventions with 90% power (β) and 5% error (α). We expected a retest rate of about 70% for the home sampling group (based on 66% in the recent CSI screening programme with home-based retest) and 50% in the clinic group [6].

### Study period

The inclusion period for the retest implementation study was 15 March – 15 August 2011. Invitations for retesting were sent between 15 August to 15 November. The time of the first consultation was marked as ‘T0’, the time of retest as ‘T1’. Initially, invitations were sent 4–5 months after the first positive test, but to include more patients, invitation for retesting was done earlier than at 4 months for the 40 last participants included. A retest was defined as a test performed 42–245 days after the first chlamydia test in the study period. Retesting within 42 days after treatment was considered a test of cure.

### Data collection and statistical methods

The database was built using a combination of variables routinely collected at the clinic and additional information collected for the study about the timing of procedures as well as data collected in questionnaires. Data collection in the questionnaire at T0 included socio-demographic variables, such as age, gender and ethnicity (based on country of birth of invitee and of parents), as well as other background and behavioural variables (education level, history of chlamydia infection, (self-reported) STI related complaints, number of sexual partners in the previous 6 months). Reasons for non-participation at baseline, if given, were registered during the consultation by the nurses. At T1 we assessed whether the person had had any STI related self-reported symptoms/complaints since the first test, the number of (new or previous) sexual partners in the 6 months before T1 and whether his/her partner had been treated at T0.

A variable sexual risk was constructed for participants of the CRI study: a person was assumed to have been at risk for STI when he/she had sex with untreated partner(s) who were already partner(s) before treatment at baseline or when he/she acquired a new sexual partner after treatment of baseline infection. Retest rate was calculated by counting the number of persons testing again within a period of 42–245 days after first testing, per home-test and clinic-test sub-group. We also checked if participants came back to the clinic for a chlamydia test within the follow-up period without making use of the retest offer. Chlamydia positivity was defined as the number of positive tests divided by all tests performed.

Univariate and multivariate logistic regression analyses were conducted to assess associations between participation at repeat testing after 6 months (dependent variable) and socio-demographic and self-reported behavioural variables (independent variables) as well as the correlates of chlamydia positivity. Backward stepwise logistic regression analyses were performed, including all variables for retest rate, and for positivity variables with a p-value for the likelihood ratio test of < 0.2. Results are given as odds ratios (ORs) with 95% confidence intervals (CIs).

Adjusted ORs give estimated effects of each variable after adjustment for all other variables.

## Results

In the inclusion period for the study, 453 *Chlamydia trachomatis* infections were diagnosed among heterosexual clients of the STI clinic. Of this group, 282 persons were invited to participate, while 112 persons were excluded because they received treatment at their first visit. For 59 chlamydia-positive patients the clinic staff forgot to ask.

### Participation CRI and retest rate

At baseline, 216 individuals consented to participate in the study and filled in the first questionnaire (participants at T0) and 66 refused to participate (77% acceptance).

Participants were invited for retesting by email 93–174 days (median 138) after their first consultation. Fifty-five percent 63% (119/216) filled in the questionnaire at T1 and 75 of them were tested. Overall retest rate was 35% (75/216). Retest rate was significantly higher in the home group (46%, 50/109) than in the clinic group (23%, 25/107, p < 0.001) (See Figure 1 and Table 1). Men were less often retested than women (15% versus 43%, p < 0.001; Table 1). Other demographic variables such as age, ethnicity, and educational level did not determine repeat testing rate. Retest rate was associated with sexual behavioural risk factors at T0: retest rates were lower in individuals with three or more partners in the 6 months before testing at T0 (24%) compared to those with one partner (38%; p = 0.07). The presence of self-reported STI-related symptoms at T0 did not influence repeat test rate. Retest rate was not associated with behavioural risk during the period since treatment: the number of sexual partners or sexual contact with a new partner in the 6 months after treatment did not influence retest rate. There was no effect on retest rate of (self-reported) symptoms since treatment at baseline, neither in men nor in women (Table 1). In multivariate analysis, including determinants available at T0 and T1, only the method of recall (home group versus clinic group) and gender were independent determinants for retesting (Table 1).

**Table 1:** Retest rates per group and determinants of repeat testing, CRI study Rotterdam 2012

				**Univariate logistic regression**			**Multivariate (n = 216)**		
	**N invited**	**n tested**	**% tested**	**OR**	**95% CI**	**p**	**OR**	**95% CI**	**p**
**Type of test offered (n = 216)**									
Test-kit by post	109	50	45.9	2.8	(1.5-5.0)	0.001	2.9	(1.6-5.4)	<0.01
Invitation to STI clinic	107	25	23.4	1					
**Gender (n = 216)**									
Male	65	10	15.4	1					
Female	151	65	43.0	4.2	(2.0-8.8)	<0.001	4.3	(2.0-9.4)	<0.001
**Age (n = 216)**									
15-19 years	47	15	31.9	1.0	(0.5-2.3)	0.94			ns
20-24 years	105	40	38.1	1.4	(0.7-2.6)	0.37			
25 years and above	64	20	31.3	1					
**Ethnicity (n = 216)**									
Dutch	99	38	38.4	1		0.30			ns
non-Dutch	117	37	31.6	0.7	(0.4-1.3)				
**Educational level (n = 216)**									
Low	58	16	27.6	0.8	(0.4-1.8)	0.62			ns
Medium	82	35	42.7	1.6	(0.8-3.1)	0.15			
High	76	24	31.6	1					
**CT infection previously to infection at T0 (n = 216)**									
Yes	61	26	42.6	1.6	(0.9-3.0)	0.13			
No	155	49	31.6	1					ns
**Complaints reported at T1 (n = 119)**									
Yes	47	29	61.7	1.09	(0.5-2.3)	0.83			
No	72	43	59.7	1					
**No of partners in past 6 months at T0 (n = 215)**									
0 partners	6	3	50.0	1.7	(0.3-8.9)	0.55			ns
1 partner	72	27	37.5	1					
2 partners	57	26	45.6	1.4	(0.7-2.8)	0.35			
3 or more partners	80	19	23.8	0.5	(0.3-1.0)	0.07			
**No of partners in past 6 months at T1 (n =119)**									
0 partners	7	3	42.9	0.5	(0.1-2.3)	0.35			ns
1 partner	52	32	61.5	1					
2 partners	29	19	65.5	1.2	(0.5-3.1)	0.72			
3 or more partners	31	18	58.1	0.9	(0.4-2.1)	0.75			
**Sex with old not treated partner(s) (n = 118) T1**									
No	42	26	61.9	1					ns
Yes	41	23	56.1	0.8	(0.3-1.9)	0.59			
Not relevant, no sex with old partner	35	23	65.7	1.2	(0.5-3.0)	0.73			
**New sex partner since treatment (n = 119) T1**									
Yes	60	38	63.3	1.2	(0.6-2.5)	0.68			ns
No	59	34	57.6	1					
**Sexual Risk (n = 113) T1**									
No old or new partners	50	31	62.0	1					ns
Risk through old partners only	25	16	64.0	1.1	(0.4-3.0)	0.87			
Risk through new partners only	24	16	66.7	1.2	(0.4-3.4)	0.70			
Risk through new as well as old partners	14	7	50.0	0.6	(0.2-2.0)	0.42			

The interval for retest invitation varied from 3–5 months. There was no difference in response rate by the duration of the period between the initial diagnosis and the retest invitation (21% for 3–4 months duration; 37% for 4–5 months and 40% for 5–6 months (p = 0.14)).

### Repeat infections

We found 13 repeat infections at T1. Three of these 13 infected persons had already had a *Chlamydia trachomatis* infection before the infection at baseline. One person reported an infection since treatment of the infection at T0 but tested negative at T1. The overall chlamydia positivity rate was 17.3% (13/75) and there was no significant difference between home and clinic group (16.0% versus 20.0% p = 0.4). Chlamydia positivity in women was 18.5% (12/65) and 10.0% (1/10) in men (p = 0.52, see Table 2). We did not find a significant difference in chlamydia positivity between age-groups, however 10 out of 13 infections were in people in the age-group under 25 years. Also educational level was not significantly related with chlamydia positivity. Repeat infections were found more frequently among non-Dutch than among Dutch respondents (27.0% [10/37] versus 7.9% [3/38]; p = 0.04). Persons reporting symptoms in the period since treatment had a significantly higher risk of infection than those without (31.0% [9/29] versus 7.0% [3/43]; p = 0.007).

**Table 2:** **Positivity rates and determinants for *Chlamydia trachomatis *repeat infections, CRI study Rotterdam 2012**

				**Univariate logistic regression**			**Multivariate (n = 72)**		
	**N Tested**	**n pos**	**% pos**	**OR**	**95% CI**	**p**	**OR**	**95% CI**	**p**
**Type of test offered (n = 75)**									
Test-kit by post	50	8	16.0	0.8	(0.2-2.6)	0.67			
Invitation to STI clinic	25	5	20.0	1					
**Gender (n = 75)**									
Male	10	1	10.0	1					ns
Female	65	12	18.5	2.0	(0.2-17.7)	0.52			
**Age (n = 75)**									
15-19 years	15	4	26.7	2.1	(0.4-11.0)	0.40			
20-24 years	40	6	15.0	1.0	(0.2-4.5)	1.0			
25 years and above	20	3	15.0	1					
**Ethnicity (n = 75)**									
Dutch	38	3	7.9	1					ns
Non-Dutch	37	10	27.0	4.3	(10.8-17.3)	0.04			
**Educational level (n = 75)**									
Low	16	3	18.8	5.3	(0.5-56.4)	0.17			ns
Medium	35	9	25.7	8.0	(0.9-67.7)	0.06			
High	24	1	4.2	1					
**Age at first sex (n = 75)**									
< 16 years	37	5	13.5	0.9	(0.1-5.1)	0.87			
16-17 years	25	6	24.0	1.7	(0.3-10.1)	0.54			
18 years and above	13	2	15.4	1					
**CT infection previously to infection at T0 (n = 75)**									
Yes	26	3	11.5	0.5	(0.1-2.0)	0.34			
No	49	10	20.4	1					
Complaints reported at T1 (n = 72)*									
Complaints yes	29	9	31.0	6.0	(1.5-24.6)	0.01	1.61	(1.6-51.2)	0.01
Complaints no	43	3	7.0	1			1		
No of partners in past 6 months at T1 (n = 72)*									
0 partners	3	0	0.0	0	(0.0-0.0)	0.99			ns
1 partner	32	3	9.4	1					
2 partners	19	4	21.1	2.6	(0.5-13.0)	0.25			
3 or more partners	18	5	27.8	3.7	(0.8-17.9)	0.10			
**Ethnicity sex partner T1 (n = 54)**									
Concordant (NL/NL)	20	3	15.0	1					
Discordant (NL/non-NL)	14	2	14.3	0.9	(0.1-6.5)	0.95			
Concordant (non-NL/non-NL)	20	6	30.0	2.4	(0.5-11.5)	0.26			
Sex with previous - untreated - partner; T1 (n = 72)*									
No	26	2	7.7	1					ns
Yes	23	4	17.4	2.5	(0.4-15.3)	0.31			
Not relevant, no sex with previous partner	23	6	26.1	4.2	(0.8-23.6)	0.10			
New sex partner since treatment T1 (n = 72)*									
Yes	38	9	23.7	3.2	(0.8-13.0)	0.10			ns
No	34	3	8.8	1					
Sexual Risk (n = 70)*									
No old or new partners	31	1	3.2	1					ns
Risk through old partners only	16	2	12.5	4.3	(0.4-15.3)	0.25			
Risk through new partners only	16	5	31.3	13.6	(1.5-130.1)	0.02			
Risk through new as well as old partners	7	2	28.6	12.0	(0.9-158.4)	0.06			

* Denominators of parameters are less than 75, due to the fact that not all respondents answered these questions.

Although we did not find significant differences (due to the small number of infections), the risk of reinfection seemed to depend on partner treatment at baseline (T0) and sexual contacts reported at T0 and between T0 and T1. Persons with a new sex partner since treatment had a higher repeat-infection rate (23.7% [9/38]) than persons without a new partner (8.8% [3/34]; p = 0.10), and rates increased with the number of reported partners in the 6 months before retesting: 9.4% for one; 21.1% for two and 27.8% for three or more partners (ns).

The rate of repeat infection was lower among those who had sex with a previous partner who was treated at baseline (7.7% [2/26]) than those with an untreated partner (17.4% [4/23]). Although the variable sexual risk could not be calculated for all tested persons, it shows that persons who only had sex with a new partner were at the highest risk for repeat infections (31.3% [5/16]), followed by those having sex with both new and old partners (28.6% [2/7]).

### Additional findings

Among the 66 persons who did not consent to participate in the study at T0, the following reasons for non-participation were reported by the nurse: 11 intended to move away, 9 did not have time, 9 did not consider the retest necessary thought not to have a reason for retesting, 8 did not want to have a testkit sent home, 6 wanted to make an appointment for testing themselves, 2 stated not to have email, 10 had other reasons and for 11 the reason was unknown.

The non-response questionnaire at T1 was sent to all CRI-participants who did not make use of the retest offered within the study. In a few persons, testing overlapped with receiving the non-response questionnaire and they were excluded from analysis. Twenty percent of non-responders (28/141) answered the questionnaire. Fourteen persons gave lack of time as a reason for not testing, three had already been tested again since the previous treatment, two did not have a new sex partner, and nine had other reasons. When asked about their opinion on the testing recall method, the non-responders in the clinic group (n = 24) mentioned lack of time (n = 9), preference to receive a testkit at home (n = 9), that they had forgotten (n = 5) or other reasons (n = 5) while the non-responders in the home group (n = 4) mentioned various non-specific reasons for not using the test kit.

Among 141 non-respondents who initially consented to participate in the retest study, 26 (18%) tested out of own initiative in our clinic: 22% (18/82) from the clinic group and 14% (8/59) from the home group. Six among these 26 (23%) were chlamydia positive.

## Discussion

In this prospective trial we compared two methods of recall for retesting of chlamydia infected heterosexual visitors of a Dutch STI clinic. We found 17.3% repeated infections, which is higher than the 12.4% baseline *Chlamydia trachomatis* infections. The overall retest rate was 35%; a higher retest rate was achieved by active recall for home-based repeat testing (46%) than recall for repeat testing at the STI clinic (23%). Females were more likely to participate in the retest.

### Strengths and limitations

To our knowledge, this is the first European prospective study investigating the effect of systematic recall on retesting and positivity rates at an STI clinic comparing home-based versus clinic-based testing. Several recall studies only report retesting rates and not positivity rates [11,12]. A disparity between the home-based and clinic-based test group was that we sent an additional reminder to those who had received a testkit at home and did not use it, thereby introducing a bias favouring participation in the home-based test group. Excluding the eight persons who responded after having received the reminder, the participation rate was 35%, which is still higher than the clinic-based group (23%; p = 0.016).

Our study was seriously limited by the time-frame within which it had to be carried out, which made it necessary to send part of the retest invitations earlier than 4 months after first treatment. This impaired the comparability to other studies and may have affected the assessment of determinants for repeat infection. We did not however find a significant difference in response rate by the duration of the period between the initial diagnosis and the retest invitation and our retest interval is still in line with a recent modelling study that showed that people were at a high risk of becoming repeatedly infected 2–5 months after treatment [13].

Our findings are also limited by the exclusion of patients who were treated at first consultation because of urethritis or because they were notified by their partner. Including them in the study seemed not to be feasible, but the proportion of chlamydia cases treated at first consultation was higher than expected beforehand. Therefore the effect of the presence of complaints at T0 on retesting within the study population is limited to persons with self-reported complaints like vaginal discharge, itching, dysuria without leucocyturia and might therefore be lower than in reality.

Another limitation of our trial was that we did not have a randomised control group with standard care only. Consequently we do not know whether doing an intervention is more effective than only mentioning retesting, nor do we know whether by any of our interventions we detected more infections than without intervention. We assessed the retest rate in non participants to explore this. During our study period, the proportion of *Chlamydia trachomatis* infected clients who did not participate in the CRI study but did return for a retest at the STI clinic on their own initiative within 42–245 days was 19% (45/237): 15% (17/112) in the group treated at first consultation 12% (8/66) in the subgroup refusing to take part in the study at T0 and 34% (20/59) in the subgroup in which the clinic staff forgot to ask for consent to participate in the study. The positivity rate in those 45 persons was 22% (10/45). Although there may have been a bias because it was not a randomized control group, our findings suggest that the retest rate with active recall is higher than without recall (35% versus 19%), while the positivity in the recall group may be somewhat lower (17% versus 22%), probably due to ‘self-triage’ as a result of new sexual risk or presence of STI-related complaints.

### Comparison with other studies

Our results are in line with a recent retrospective study based on laboratory data from the Netherlands which found similarly high positivity rates after 3–12 months among patients in the STI clinic (19.4%), at gynaecologists (14.8%) or GPs (17.4%) [9]. The determinants for reinfections we found are known risk factors for *Chlamydia trachomatis* infections: young age, non-Dutch ethnicity, low education [3-6,14,15].

We do not know if persons are commonly retested at the GP, although we expect it may be a barrier that they have to pay for STI-tests at the GP within their own-risk payment in health care insurance whereas in the Dutch STI clinics STI-tests are provided for free. In the recent retrospective Dutch study, rescreening rates were similar for the STI clinic (33.4%) and gynaecologists (30.3%) but lower for general practitioners (23.0%) [9]. As no practical guidelines on retesting are in place in the Netherlands, retests are likely to be only offered to a group of higher-risk cases selected by provider triage or self-triage, as the authors suggest.

Our results are based on a high-risk STI clinic population in a large, multi-ethnic city. Retest rate as well as positivity may well differ in other areas or populations. The retest rate in our study was lower than in a previous Chlamydia Screening Implementation (CSI) study [6] which achieved a retest rate of 66% . A difference with the CSI study is that the participants in were already used to home-testing and had chosen, by participating, to receive a testkit at home and receive related information via the internet; in addition, two automatic reminders were sent to non-responders in CSI versus one in our study and CSI made use of an official website with specific information. The participants in our study had no previous experience with home-based testing and may have been in favour of coming to the clinic, although the test rate among those who could come to the clinic at a convenient time was relatively low. Retest rates of 21-27% were achieved in other studies with mailed home-test kits when retesting was done after 3 months or more [16,17]. Often higher retest rates (up to 48%) are reported in the period <3 months, tests which may be tests of cure [17].

### Interpretation

Despite the limitations, we could show that - as expected - repeated infections are a common problem in chlamydia patients. STI clinics should provide retesting advice and offer retesting in a more proactive way to *Chlamydia trachomatis* infected individuals.

To achieve higher retest rates, combined strategies may be required. Among patients using mailed test kits and telephone reminders in the US, the re-screening rates were 59.2% at family planning clinics and 43.5% at STI clinics [17]. In a meta-analysis, it was found that mailing testkits is an effective strategy to increase rescreening, but also that telephone reminders are promising. Text messaging (SMS) has been found to have similar effects as letters and phone calls, but is much cheaper and easier to implement [10].

Although the home-based testkit achieved the highest participation rate in our study, the majority of kits was not used and the question remains whether this method is cost-effective. Sending invitations, testkits and reminders manually is time-consuming and is possibly not achievable, logistically or financially, in STI clinic settings. Even without home-based testkits, we found that patients frequently came for a retest, also on their own initiative. Offering a retest and reminders by SMS may be more feasible.

Recently, two studies in Australia demonstrated a moderate increase in repeat testing by sending a short message reminder, although combining SMS-text-messages with a financial incentive did not have further impact [12]. This impact may be influenced by the reason for testing and type of relationship of patients. In MSM who are more familiar with repeat STIs testing, reminders by SMS have been shown to double the repeat test rate [18]. Challenges in using SMS reminders are dealing with undeliverable messages and ensuring that health professionals actually send them to the selected patients [11,12]. Automated SMS reminders should preferably be built-in to electronic patient registration systems. It has been demonstrated in various interventions that patients may choose different strategies depending on their personal circumstances [10], hence personal preferences should be taken into account. However, providing good and accessible retest services is not the only way to reach a higher retest rate. Effective sexual health education that seeks to change attitudes to testing and risk perception among young people at risk for chlamydia is crucial. The accessibility of health services, low-risk perception, fear and worry were described as barriers to testing in an European review on HIV testing [19]. Accessibility of health services and sufficient self- efficacy are important but not sufficient explanations [20], as self-efficacy was not a significant predictor of STI testing among adolescents [21,22] but attitudes and susceptibility were.

## Conclusions

High rates of *Chlamydia trachomatis* repeat infections indicate the need for an active offer and support of retesting and should be included in guidelines and recommendations for STI control.

Home-based testing was more effective than clinic-based testing. However, various strategies including self-triage of patients may increase repeat testing and personal preferences should be taken into account. Automated SMS reminders inviting patients for retesting at the clinic may be the most cost effective and feasible method of choice to be implemented by STI clinics. A prerequisite for strategies for retesting, like any good public health intervention, is that they are proven to be (cost)effective and can be paid for within the public health care system.

## Competing interests

The authors declare that they have no competing interests.

## Authors’ contributions

HG was the author responsible for design of the CRI study, assisted by IvdB. MW analysed CRI data in cooperation with HG, HG analysed STI clinic data. AL coordinated the laboratory analysis and assisted in logistic of testkits. All authors have commented on draft versions and approved the final version.

## Pre-publication history

The pre-publication history for this paper can be accessed here:

http://www.biomedcentral.com/1471-2334/13/239/prepub
